# Mouthguard types, properties and influence on performance in sport activities: a narrative review

**DOI:** 10.3389/fmed.2025.1527621

**Published:** 2025-01-28

**Authors:** Ke Wang, Yitong Liu, Zihe Zhao, Shengjie Zhou, Min Zhang

**Affiliations:** ^1^Department of Implantology, Shanghai Engineering Research Center of Tooth Restoration and Regeneration, Tongji Research Institute of Stomatology, Stomatological Hospital and Dental School, Tongji University, Shanghai, China; ^2^HKU Business School, University of Hong Kong, Hong Kong, Hong Kong SAR, China

**Keywords:** mouthguards, history, sport, types, properties, athletic performance

## Abstract

Mouthguards are proven devices placed inside the mouth to prevent oral lacerations, dental injuries, and jaw fractures. Endorsed by the World Dental Federation, mouthguards are crucial for preventing orofacial and dental trauma. However, their adoption in sports is challenged by limited guidance, communication barriers, and cost considerations. Based on extensive literature research in PubMed/MEDLINE, this narrative review summarizes the historical development of mouthguards, elaborates on their primary classifications, and examines the factors influencing their properties. Importantly, the impact of mouthguards on sports performance is clarified in this study. Overall, using mouthguards in sports not only reduces the occurrence and severity of dental injuries but also holds promise for enhancing athletic performances such as strength, aerobic capacity, agility, balance, and flexibility. Therefore, promoting mouthguard use in the sports community should be encouraged.

## 1 Introduction

Sports-related accidents are a significant health issue, substantially impacting daily life. Nearly one-third of orofacial and dental injuries result from sports safety accidents, leading to considerable financial burdens for treatment (1). A previous study reported that orofacial and dental injuries affect nearly a billion people and incur annual treatment costs exceeding 5 million dollars (2). Additionally, as many as 52% of children aged 11–13 have experienced oral injuries due to sports, and more than 70% of athletes have sustained orofacial and dental injuries (3). Sports-induced orofacial and dental injuries include pulpal necrosis, intradental resorption, crown fractures, soft tissue injuries, subluxations, and maxillary fractures (4). These injuries can disrupt an athlete’s participation and training schedules, incur high treatment costs, and result in enduring cosmetic issues (5).

Mouthguards play a critical role in mitigating sports-related injuries to the oral and maxillofacial region by absorbing and distributing impact forces, thereby lessening the force transmitted to dental hard tissues, mandibular condyles, and articular disks (6). Due to limited centralized monitoring data, no large-scale studies currently explore the influence of mouthguards on oral and maxillofacial injuries. It has been observed that the risk of orofacial trauma in contact sports increases by 1.6–1.9 times when a mouthguard is not used (7). Furthermore, incorporating mouthguards has been shown to decrease the transmission of impact energy to targeted head regions, potentially lessening the severity of traumatic brain injuries (8). The broader role of mouthguards in safeguarding against brain injury is supported by current literature, which highlights their effectiveness in sports where head impacts are prevalent (3, 9). The World Dental Federation endorses the use of mouthguards as a preventive approach against orofacial and dental injuries, with a particular recommendation for custom-made mouthguards crafted by dental professionals (10). However, several factors hinder the adoption of mouthguards by athletes, including inadequate coaching guidance, communication limitations, and perceived impacts on athletic performance (11). The financial aspect of mouthguard acquisition also influences athletes’ decision-making processes. Beginning with the history of mouthguards, this study aims to review the main types of mouthguards and the potential factors measuring their performance, with a special focus on the effect of mouthguards on sports performance.

## 2 Search criteria

As a narrative review, this study performed a comprehensive literature search using mouthguard as a search term, focusing on studies that are directly pertinent to the use of mouthguards in athletics, encompassing their classification, characteristics, and implications for athletic performance. Peer-reviewed systematic reviews, cohort studies, randomized controlled trials, and case-control studies written in English were included, with no restrictions on article type or geography. Non-empirical works such as editorials, commentaries, and non-systematic reviews, as well as studies not aligned with the practical applications of mouthguards in sports or clinical settings, were excluded.

The literature was obtained by extracting data from databases through the review of titles and abstracts. Full texts of studies deemed relevant were retrieved. Since this study is a narrative review, it did not strictly adhere to the systematic review methodology prescribed by the Preferred Reporting Items for Systematic Reviews and Meta-Analyses guidelines.

## 3 History of mouthguards

Boxing, as the pioneer in the use of mouthguards, has a history that dates back to the late 19th century. During this period, boxers crafted makeshift mouthguards from materials like cotton, tape, sponge, or small wooden pieces. These early mouthguards were clenched between the teeth to offer basic protection against the sport’s forceful impacts. The 1930s marked a pivotal shift in mouthguard technology with the advent of custom-made mouthguards. Dentists began using dental impressions, wax, and rubber to create mouthguards that were more comfortable and provided enhanced shock absorption and protection (7). Rugby, another sport with a high risk of oral and facial injuries, soon followed boxing’s lead in adopting mouthguards. In the 1950s, dental injuries were reported to account for most injuries in rugby, ranging from 23 to 54% (12). This prompted the American Dental Association (ADA) to endorse latex mouthguards in rugby and other contact sports by 1960. Currently, the ADA advocate for the application of mouthguards in 30 sports or exercise activities with a high risk of orofacial injury (13).

Over the years, the design and materials of mouthguards have evolved significantly. Modern mouthguards are crafted from advanced materials such as ethylene-vinyl acetate (EVA), polyurethane, and silicone, each offering unique properties to enhance comfort, fit, and protective capabilities. The ongoing development of mouthguard technology focuses on enhancing shock absorption, optimizing material properties, and improving fit through advanced manufacturing techniques. The evolution of mouthguards from simple, makeshift devices to sophisticated, personalized equipment highlights the growing recognition of sports safety and the role of dental technology in enhancing athletic performance.

## 4 Types of mouthguards

**FIGURE 1:**
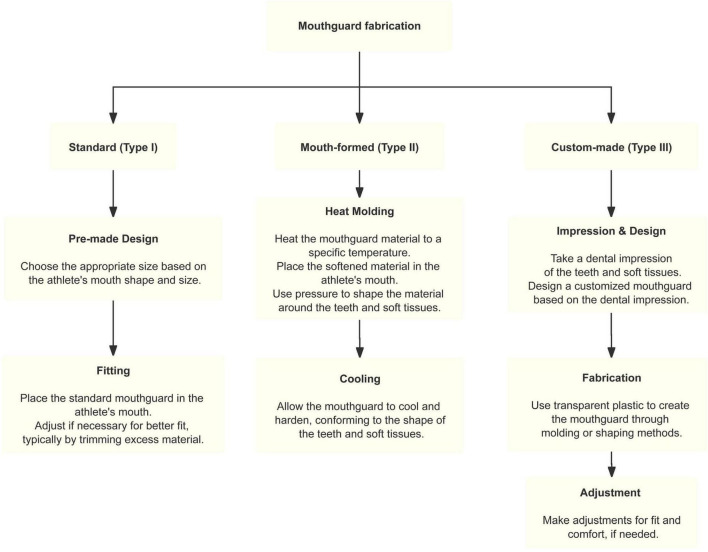
The fabrication process of these types of mouthguards.

Mouthguards are defined as removable devices designed to decrease the risk of oral trauma and protect dental structures (14). The American Dental Association’s Standard Specification for Dental Healthcare Products categorizes mouthguards into three primary types: standard (Type I), mouth-formed (Type II), and custom-made (Type III) (10). Figure 1 illustrates the fabrication process of these types of mouthguards, and Table 1 summarizes the materials, fabrication methods, and properties of each type.

**TABLE 1:** Materials, fabrication methods, and properties of mouthguards.

Type of mouthguard	Materials	Fabrication methods	Properties
Type I (Standard)	Polyurethane, vinyl acetate copolymers	Prefabricated	Cost-effective, minimal protection, poor comfort and fit
Type II (Mouth-formed)	Thermoplastic materials, chemically shaped varieties	Heat-softened and molded	Enhanced stability and adaptability, may affect ventilation and thickness during molding
Type III (Custom-made)	Ethylene-vinyl acetate (EVA), polyurethane, acrylic resin	Custom-fabricated using dental impressions or 3D printing	Superior comfort and fit, optimal protection for both soft and hard tissues, reduced gag reflex symptoms, maintains oral moisture levels

Standard mouthguards are prefabricated and non-customized devices, typically featuring a U-shaped design with a central groove to accommodate teeth (15). These mouthguards are designed to be clenched for fit, rather than providing a tailored fit. Despite their initial popularity owing to cost-effectiveness, standard mouthguards exhibit suboptimal comfort and universal applicability due to variances in dental arch sizes among demographics (16). Meanwhile, standard mouthguards offer minimal protection to oral and maxillofacial tissues, making this type of mouthguard the least desirable option. Wearing a standard mouthguard can cause athletes to experience several uncomfortable symptoms, such as difficulty breathing, nausea, impaired speech, and distraction. Studies have shown that the occurrence of these symptoms is markedly decreased with the use of mouth-formed and custom-made mouthguards (17).

Type II mouth-formed mouthguards are currently the most widely used mouthguards among athletes, including thermoplastic and chemically shaped varieties, with thermoplastic being the most common (15). This type of mouthguard is heat-softened and placed in the mouth to adapt to an individual’s intraoral anatomy. Compared to standard mouthguards, mouth-formed mouthguards offer enhanced stability and adaptability to the intraoral anatomy (18). However, existing literature also reports some shortcomings of mouth-formed mouthguards (19, 20). For example, the use of mouth-formed mouthguards may exert potential impacts on ventilation and a decrease in thickness during the molding process, which can compromise their protective capabilities (19). Additionally, researches have shown that some of the materials utilized in this type of mouthguard have a wide thermoplasticity and temperature range required for intraoral operation (20). In addition, the standards of the American Dental Association also include chemically shaped mouthguards, but there are currently commercial mouthguards are yet to emerge (21).

Custom-made mouthguards (type III), fabricated by dental professionals based on intraoral models, have garnered traction in recent years (15). These mouthguards offer superior comfort, reduce gag reflex symptoms, and help maintain oral health by preserving moisture levels (22). Compared to mouth-formed and standard mouthguards, custom-made mouthguards provide comprehensive coverage for both dentition and soft tissues, with appropriate thickness and extension into the oral cavity’s vestibule, thus enhancing protection for the soft and hard tissues in the oral and maxillofacial region (23). Furthermore, in recent years, the advent of digital technology has allowed for the direct 3D printing of this type of mouthguard, ensuring a more precise fit to the oral tissues of athletes and further improving the efficacy in preventing trauma (24).

## 5 Factors for measuring mouthguard performance

The efficacy of mouthguards is assessed through a range of physical properties, including shock absorption capacity, hardness, tear strength, stiffness, water absorption and tensile strength (25). These properties are integral when selecting the mouthguards, as they collectively determine the protective capabilities, durability, and stability of mouthguard in the oral cavity. Specifically, shock absorption, hardness, and stiffness are key to evaluating the protective capacity, while tensile strength and tear strength indicate longevity. In addition, water absorption reflects how well the mouthguard will maintain its integrity in the moist oral cavity.

Research indicates that wearing a mouthguard made from various materials can significantly decrease the likelihood of tooth fractures under constant force and increase the force threshold required for tooth fracture (26). This reduction in transmitted external force is essential for protecting the teeth and the maxillofacial region from impact. The original material used for mouthguards was primarily latex rubber; however, advancements in materials science have introduced a variety of options, including ethylene-vinyl acetate (EVA) copolymer, polyvinyl chloride, acrylic resin, and polyurethane. EVA copolymer is currently the most widely used material for custom mouthguards. However, it is important to note that material properties can be tailored to specific needs, meaning no single material holds a distinct advantage over others. The choice of material should be based on the unique characteristics of the sport and the anatomical features of the oral structures, with careful consideration given to shock-absorbing capacity and stiffness to ensure effective protection against both hard and soft impacts.

The protective efficacy of a mouthguard is influenced by several factors, with labial thickness being particularly significant. Studies suggest that the shock-absorbing capacity of mouthguards increases with labial thickness up to a point, but additional thickness does not provide extra protection when the thickness exceeds 4 mm (27). Specifically, the thickness should be 3–4 mm on the labial surface of the central incisors, 2–3 mm on the occlusal surface of the posterior teeth, 4 mm on the incisal edge of the anterior teeth, and 1 mm on the palatal side. Regarding coverage, the labial extension should be 2 mm short of the vestibular reflection with a rounded cross-section, while the palatal extension should extend just beyond the cervical margin of the palatal surface of the teeth with a tapered cross-section (28). The type of mouthguard is another important consideration, as the degree of protection can vary among different custom-made mouthguards (29). The occlusal support area also influences the protective effect of mouthguards; larger occlusal areas can reduce the impact transmitted to the mandibular boundary and displacement, thereby decreasing the risk of mandibular deformation and fractures (30).

## 6 The impact of mouthguards on athletic performance

Despite the proven protective effects of mouthguards against orofacial and maxillofacial injuries, there is currently no consensus in the literature regarding whether wearing mouthguards impacts athletic performance (31). Common indicators for assessing athletic performance include breathing, strength, balance, agility, flexibility, and performance in specific sports. The types of mouthguards utilized in studies vary, with custom-made mouthguards being the most extensively studied, followed by mouth-formed mouthguards, and fewer studies focusing on standard mouthguards (32). The general consensus is that a properly designed mouthguard does not negatively affect sports performance (33).

Strength is a critical factor in many sports, particularly in contact sports where force exertion is vital for competitive performance (34). When athletes wear mouthguards, the inherent thickness of these devices may influence muscle activity and force generation by altering the vertical dimension of occlusion (VDO) and the positioning of the temporomandibular joint (TMJ). The VDO is defined as the interocclusal distance between the maxillary and mandibular arches when the teeth are in maximum intercuspation (35). The impact of varying VDO magnitudes remains unclear and appears to differ among individuals (36). Although an increase in VDO and the adjustment of TMJ positioning are theorized to refine muscle activation patterns and potentially enhance force output, further research is necessary to delineate the mechanisms involved and their practical applications in athletic performance (37). Moreover, while some studies report improvements in dynamic strength, isometric force, and cardiopulmonary parameters for athletes wearing mouthguards, it remains unclear whether these findings are applicable to all athletes in different sports environments (38). The concurrent activation potentiation (CAP) phenomenon, where isometric contraction of one part of the body enhances muscle strength in another, may also contribute to improved athletic performance with mouthguards. A previous study conducted by Ebben er al demonstrated that when wearing a mouthguard and clenching the jaw, the CAP effect may be facilitated, thereby enhancing strength performance (37).

Aerobic capacity is another significant consideration for athletic performance, especially in endurance and high-intensity interval training sports. Some aerobic exercise indicators, such as tidal volume and maximum oxygen uptake, have been reported in the literature as potentially being negatively affected when wearing a mouthguard (31). Concerns have been raised that wearing a mouthguard may restrict oral airflow and affect breathing efficiency, leading to resistance to its use (39). This concern arises from the mouthguard acting as a physical barrier that could reduce oral ventilation, particularly during high-intensity exercise when athletes require greater pulmonary ventilation to meet oxygen demands. Regarding oxygen uptake, maximal oxygen uptake serves as a critical metric for assessing aerobic exercise capacity (40). A study by Terence et al. (41) suggests that while wearing a mouthguard may impose some limitations on oxygen uptake, this impact may not be significant in actual sports performance. Furthermore, the negative impact on aerobic parameters may be related to the type of mouthguard. Custom-made mouthguards have shown significant advantages over the other two types in enhancing athletic performance. This type of mouthguard is more adaptable and comfortable, with less interference with breathing and oral moisture. Consequently, wearing custom-made mouthguards has not been reported to negatively affect aerobic capacity compared to other types of mouthguards (39). In summary, existing scientific research does not support the notion that mouthguards significantly negatively impact aerobic performance. In summary, existing scientific research supports the notion that custom-made mouthguards typically maintain high respiratory efficiency and oxygen intake while protecting the athlete’s oral cavity (42).

Agility, balance, and flexibility are three key dimensions of an athlete’s comprehensive athletic ability. Agility, which involves an athlete’s ability to quickly change direction in a short time, is crucial in sports that require rapid reactions, such as soccer, basketball, and rugby. Balance pertains to an athlete’s ability to maintain a stable posture during movement, which is essential for sports that require standing on one foot or performing skillful movements on unstable surfaces. Flexibility, which describes the range of motion of an athlete’s joints, is particularly important in sports that involve large-scale body extension, such as gymnastics and dance (32). These indicators are essential for athletes to respond, maintain stable postures, and make diverse physical adjustments in a rapidly changing competitive environment. Nam et al. (43) further investigated the influence of a customized mouthguard on body alignment and balance performance in professional basketball players. Their findings showed that wearing a customized mouthguard positively affects balance performance. A study by Ebben et al. found that athletes wearing custom-made mouthguards demonstrated improved jump height and rate of force development in vertical jump tests, which may relate to the mouthguard enhancing muscle activity through the CAP effect (44). Golem and Arent’s research found that jaw repositioning techniques may improve athletic performance by enhancing postural control and spinal alignment; however, over-the-counter jaw repositioning mouthguards did not show significant effects on agility, balance, and flexibility in college-aged male athletes (34). Additionally, the impact of mouthguards on athletic ability is related to the athlete’s familiarity with the mouthguard (32). As athletes become accustomed to wearing mouthguards, they may learn to adjust their movements, thereby reducing any potential negative impacts on athletic performance. In summary, mouthguards not only protect the teeth but also typically enhance athletic ability (37).

## 7 Highlight

The present study examines the history, classification, and properties of the mouthguards, as well as their potential impacts on athletic performance. It highlights the properties of mouthguards, from standard design to custom-made type, which offer superior protection and potential performance benefits. This study demonstrates the role of mouthguards in reducing the risk of oral and maxillofacial injuries in sports, along with the evidence regarding their potential impact on athletic performance. Fostering greater awareness and acceptance of mouthguards among athletes is essential, further research is necessary to elucidate the mechanisms by which mouthguards may enhance athletic performance and to develop even more effective designs.

## 8 Clinical implication

Clinicians should advocate for the use of mouthguards among athletes, particularly custom-made types, as they offer both protection and potential performance enhancements for specific physical actions. It is essential to educate athletes about the benefits of mouthguards in preventing sports-related oral injuries, ensuring that the advantages of these devices are fully recognized, leading to safer and more effective athletic pursuits. Furthermore, a comprehensive understanding of factors influencing mouthguard properties can assist clinicians in designing the most appropriate mouthguards.

## 9 Conclusion

In conclusion, the incorporation of mouthguards in sports is strongly encouraged due to their multifaceted benefits. Their utility extends beyond the prevention of orofacial and dental injuries as they also hold the potential to enhance various parameters of athletic performance. Custom-made mouthguards provide distinct advantages over standard and mouth-formed alternatives. These mouthguards not only offer enhanced protection but may also positively influence sports performance through mechanisms such as altered TMJ positioning, increased VDO, and the facilitation of CAP via jaw clenching. The strategic integration of mouthguards into sports equipment is a crucial step toward optimizing both the health and performance of athletes in sports.
